# Is Participation in Organized Leisure-Time Activities Associated with School Performance in Adolescence?

**DOI:** 10.1371/journal.pone.0153276

**Published:** 2016-04-13

**Authors:** Petr Badura, Erik Sigmund, Andrea Madarasova Geckova, Dagmar Sigmundova, Jan Sirucek, Jitse P. van Dijk, Sijmen A. Reijneveld

**Affiliations:** 1 Institute of Active Lifestyle, Faculty of Physical Culture, Palacky University, Olomouc, Czech Republic; 2 Olomouc University for Society and Health Institute, Palacky University, Olomouc, Czech Republic; 3 Department of Health Psychology, Faculty of Medicine, Safarik University, Kosice, Slovakia; 4 Graduate School Kosice Institute for Society and Health, Safarik University, Kosice, Slovakia; 5 Institute for Research of Children, Youth and Family, Faculty of Social Studies, Masaryk University, Brno, Czech Republic; 6 Department of Community and Occupational Medicine, University Medical Center Groningen, University of Groningen, Groningen, the Netherlands; University of Tennessee Health Science Center, UNITED STATES

## Abstract

**Background:**

Organized leisure-time activities (OLTA) have been identified as a context suitable for improvement of school performance. This study aimed to assess the associations between participation in OLTA and school engagement, school-related stress, academic achievement and whether these associations differ by specific pattern of OLTA participation, gender and age. Furthermore, it assessed whether OLTA participants are more likely to acquire support for schoolwork from outside the family.

**Methods:**

The sample concerned 10,483 adolescents (49.2% boys) aged 11, 13 and 15 from the Health Behaviour in School-aged Children data collection in 2014 in the Czech Republic. Logistic regressions adjusted for gender and age were used to analyse the associations between participation in OLTA and four education-related outcomes.

**Results:**

Participation in OLTA was associated with higher school engagement, lower levels of school-related stress and better academic achievement regardless of gender and age. The strongest associations were observed for adolescents involved in various types of OLTA concurrently, with odds ratios ranging from 1.34 (95% confidence interval (CI) 1.17–1.54) for lower school-related stress to 1.97 (95% CI 1.73–2.25) for above-average academic achievement. OLTA participants were also more likely to have a non-familial person to help them with schoolwork, though this association was weaker in 15-year-olds.

**Conclusion:**

Youth involvement in OLTA is linked to general better school performance and attachment to school. Adolescents participating in more activities at the same time have the best school performance.

## Introduction

School is an essential developmental context during adolescence [1] and closely relates to health of young people. On the one hand, better school performance and higher school attachment are predictive not only of school completion [2] and future socioeconomic success [3], but there is also a link to enhanced health and well-being in adolescents [4–6]. Greater levels of school-related stress, on the other hand, are detrimental to health [7].

Organized leisure-time activities (OLTA) have been proposed as a potential “booster” of education-related outcomes [8, 9]. Numerous studies have confirmed that participation in OLTA is associated with improved academic achievement [10–12] or higher school engagement [8, 13, 14] and have documented the stress-buffering effects of OLTA [15]. It is assumed that OLTA fosters initiative [16], non-cognitive skills [17], learning goal orientation [18] and the formation of supportive social networks [1]. This can in turn lead to better achievement and attachment to school. Moreover, those who participate in OLTA can form and maintain stronger relationships both with their peers and adults outside their family [19, 20]. These can then assist them in overcoming a potential lack of ‘educational resources’ within their own family.

As distinct OLTA are unique in their contents and the developmental assets they offer to youth, they may also yield different education-related effects [11, 17, 21]. For instance, Himelfarb et al. [12] have observed improved school performance in adolescents attending art activities, clubs or sports, while membership to religious groups was unrelated to grades. In addition to the activity type itself, it has also been suggested that involvement of adolescents in more contexts (i.e. various activities at the same time) is better for their development [9, 22–24]. However, a certain saturation effect may occur, i.e. a plateau or even a slight decline in positive education-related outcomes when the number of activities is too high [10, 14, 25].

Hitherto, research on this topic has been conducted only scarcely in Europe and has mostly originated from the USA. Considering the differences of educational and OLTA systems across cultures it is desirable to focus on this issue in the European context as well. The present study, therefore, aims to assess whether participation in OLTA is associated with improved school performance in Czech adolescents and whether the associations differ by specific pattern of OLTA participation, gender and age. Furthermore, it aims to explore whether OLTA participants are more likely to acquire support for schoolwork outside the family.

## Methods

### Participants

Data analysed in the present study were drawn from the 2013/2014 Health Behaviour in School-Aged Children (HBSC) study in the Czech Republic. The HBSC study is a large-scale cross-national study carried out in four-year intervals in 43 countries in Europe and North America. It investigates health behaviours and their determinants and consequences in 11-, 13-, and 15-year-olds [26].

Schools were the primary sampling unit and were selected randomly, after stratification by region and type of school (primary vs. secondary schools), from the database of the Czech Ministry of Education, Youth and Sports. We received consent to carry out the survey in 243 out of 244 contacted schools (response rate 99.6%). Then, one class from each of the 5^th^, 7^th^, and 9^th^ grades, which in general correspond to the age categories of 11, 13, and 15 years in the Czech education system, was selected at random per school.

Out of 16,298 pupils registered in the classes selected for the survey, we obtained questionnaires from 14,539 respondents (response rate 89.2%); 1,729 pupils were not present in school during the survey (10.6%), with the most common cause of absence being an illness and sports or academic competitions. Thirty pupils refused to fill in the questionnaire (0.2%). Next, in line with the HBSC protocol, we selected only 11-, 13- and 15-year-old adolescents from this sample (n = 10,795). Finally, we excluded 312 cases due to missing data on age, gender, all OLTA or education-related items, an excessive number of missing values on all items, or several unlikely responses throughout the questionnaire. The final sample concerned 10,483 respondents.

### Procedure

The survey was conducted between April and June 2014. The questionnaires were distributed by trained administrators. Teachers were not present in the classrooms in order to minimize the response bias. One school lesson (45 minutes) was dedicated to completing the questionnaire. The Czech HBSC study was conducted under auspices of Ministry of Education, Youth and Sports of the Czech Republic and the World Health Organization Country Office in the Czech Republic. The Czech legislation does not require written informed consent for participation in questionnaire surveys. The consent to carry out the study was obtained through school management at all the schools involved in the survey. Participation in the survey was anonymous and voluntary. Parents of the pupils were informed about the survey, its content and purpose via the school management in advance and could withdraw their child if they wished. Moreover, schools in the Czech Republic usually collect a so-called "general consent" from the pupils' parents/legal guardians at the beginning of each school year. This way of doing covers consent to take photographs or audio/video footage of pupils, provision of school counsellor's or psychologist's services, and participation in anonymous questionnaire surveys. The final decision is therefore, in principle, taken by the school administration (the school principal or employees on behalf of him/her). Prior to administration of the questionnaires the respondents were notified of the option to opt out of the study. The study design was approved by the Ethics Committee of the Faculty of Physical Culture, Palacky University in Olomouc (No. 17/2013).

There were five versions of the questionnaire—one for 11-year-olds, two for 13-year-olds, and two for 15-year-olds. The versions for 13-year-olds contained more questions than the one for 11-year-olds, and the versions for 15-year-olds still more. Versions for all ages comprised an identical set of mandatory questions, but they differed regarding the optional items, with two different versions for the two oldest age categories. The two distinct versions of questionnaires for both the older age categories were used with the intent to include as many areas of interest as possible while bearing in mind the age-appropriate length of the questionnaire. The item on school support outside the family was offered only to half of the 13-year-olds and 15-year-olds, with a total of 3,563 respondents.

### Measures

Participation in OLTA was measured using the question ‘*In your free time*, *do you do any of these organized activities*?’ with dichotomous response *yes/no* [27]. We investigated six particular types of organized leisure-time activities. The activities included *team sports* (e.g. football, basketball, volleyball), *individual sports* (e.g. tennis, gymnastics, karate), *art school* (e.g. music instruments, singing, dance, drama), *youth organizations* (e.g. Scouts, Sokol), *leisure centres or after-school clubs* (e.g. chess, model building, debate clubs), and *church meeting/singing* (e.g. Salesian choir). These were selected based on four most common groups of activities suggested by Lerner [28] and supplemented by church meeting/singing given the previously shown importance of religiosity in this part of Europe [29]. In addition, we used two categories of sports (team and individual sports) as they offer different developmental experiences to youth [30]. Missing answers were considered to represent ‘no’ unless all six OLTA items were missing. Then the respondent was excluded, as indicated above (*n* = 252).

Next, the respondents were split into five groups of OLTA participation patterns based on the cluster analysis, which was carried out using the similar procedure as in the previous study on OLTA [31]. ‘Inactive’ adolescents (*n* = 1,967) did not participate in any OLTA. ‘Team sports’ participants (*n* = 1,929) were engaged only in team sports. The cluster ‘individual sports’ (*n* = 1,371) comprised adolescents doing individual sports and slightly more than a half of them participated at the same time in team sports. Over half of the ‘artists’ (*n* = 1,949) engaged in an individual and/or a team sport together with arts. ‘All-rounders’ (*n* = 3,267) included the remaining respondents, with 85% of them being involved in two or more OLTA.

School performance was assessed using three school-related items from the HBSC mandatory questionnaire [32]. School engagement was measured using the item ‘*How do you feel about school at present*?’ We dichotomized four response categories as *I like it a lot* and *I like it a bit* vs. *I don’t like it very much* and *I don’t like it at all*.

School-related stress was assessed by the item ‘*How pressured do you feel by the schoolwork you have to do*?*’* The four responses were dichotomized as not being stressed (*Not at all / A little*) vs. being stressed (*Some / A lot*).

Academic achievement was measured using the item ‘*In your opinion*, *what does your class teacher think about your performance compared to your classmates*?*’* The four response categories were dichotomized as above-average achievement (*Very good / Good*) vs. the remainder (*Average / Below average*).

The Family Affluence Scale (FAS) was used to measure the socioeconomic status of participants. The scale consists of six items investigating *car ownership*, *having one’s own bedroom*, *number of computers in the household*, *number of foreign family holidays*, *number of bathrooms*, and *dishwasher ownership* [32]. The FAS summary score was converted to a final score, which has a consistent, normal distribution ranging from 0 to 1. Then, we created tertile groups of low (0 to .333), middle (.334 to .666), and high (.667 to 1) socioeconomic status [33]. This categorised variable was used as a control variable in the logistic regression models. The validity of the FAS has been tested both at the individual level (against the parents’ reported wealth) and at the national level (against Gross Domestic Product), indicating a good validity [34]. The scale also showed a good stability over time [35].

Last, we assessed whether adolescents had someone outside their family to help them with school duties using the question ‘*Does anyone support you / help you with schoolwork*?’ with two items: *An adult outside my family* or *One of my friends*. The respondents were considered to be supported or helped with schoolwork by a person outside their family, if they answered *yes* to at least one of the items. This question was not present in the version for 11-year-olds, and we obtained 3,374 valid responses.

More detailed information on the questionnaire used in the last HBSC survey in 2013/2014 can be found in the HBSC International Protocol [32], which can be obtained at the HBSC website: http://www.hbsc.org/methods/.

### Statistical analyses

First, we described the composition of the sample, its participation in OLTA and its education-related outcomes. The statistical significance of gender and age differences with regard to participation in particular types of OLTA and education-related outcomes was assessed by chi-square tests. Second, we split the participants on the basis of the pattern of their OLTA involvement, by using cluster analysis. We obtained five distinct clusters, which was the smallest number possible, yet having reasonable intra-cluster similarity and, at the same time, low inter-cluster similarity. Third, we analysed the associations of the dichotomized overall OLTA variable (at least one activity vs. none) with school engagement, school-related stress, academic achievement and school support outside family using logistic regression analyses. For this, we assessed crude associations per education-related outcome (Model 1). Next, we adjusted for age and gender (Model 2). Last, we tested interaction effects of gender and age on the associations between participation in OLTA and the four education-related outcomes. We then repeated these analyses for the separate clusters of OLTA. Additional adjustment for socioeconomic status as measured by the FAS [31] did hardly affect findings (Tables A and B in S1 Table). The data were analysed using routine regression because multilevel analysis found no indication for clustering by school [36]. The statistical analyses were carried out using IBM SPSS 22 for Windows (IBM Corp. Released 2013).

## Results

The characteristics of the sample are presented in Table 1. In all, 81% of respondents participated in one or more OLTA (*M* = 1.51, *SD* = 1.17). Younger adolescents of both genders were involved in all the types of OLTA more often than older adolescents (*p*<0.001), with the exception of individual sports in boys. The breadth of activities (i.e. number of OLTA in which respondents participated concurrently) also decreased with increasing age, this trend being stronger for girls. Girls aged 11 years participated on average in 1.86 activities and girls aged 15 years in 1.20 activities. In boys the decrease was milder, from 1.67 activities at age 11 years to 1.24 activities at the age of 15.

**Table 1:** Description of the study population: rate of respondents’ participation in organized leisure-time activities (top part) and education-related outcomes (bottom part) by gender and age.

	Gender				Age						Total	
	Boy		Girl		11		13		15			
	(n = 5161)		(n = 5322)		(n = 3324)		(n = 3534)		(n = 3625)		(n = 10483)	
	n	%	n	%	n	%	n	%	n	%	n	%
≥1 activity	4233	82.0	4283	80.5	2895	87.1	2953	83.6	2668	73.6	8516	81.2
No activity	928	18.0	1039	19.5	429	12.9	581	16.4	957	26.4	1967	18.8
Higher school engagement	3555	69.0	4070	76.6	2591	78.0	2559	72.5	2475	68.4	7625	72.8
Low school-related stress	3649	70.8	3653	68.7	2513	75.8	2347	66.5	2442	67.4	7302	69.7
Above-average academic achievement	2754	53.5	3171	60.0	1972	59.7	1952	55.5	2001	55.4	5925	56.8
School support outside the family	542	32.5	833	48.9	*N/A*		660	40.1	715	41.3	1375	40.8

% represents the percentage of respondents within the column concerned (gender, age, total); the item on school support outside family was asked for only in one of both questionnaire versions for 13-year-olds and for 15-year-olds (total n = 3,374).

**Fig 1:**
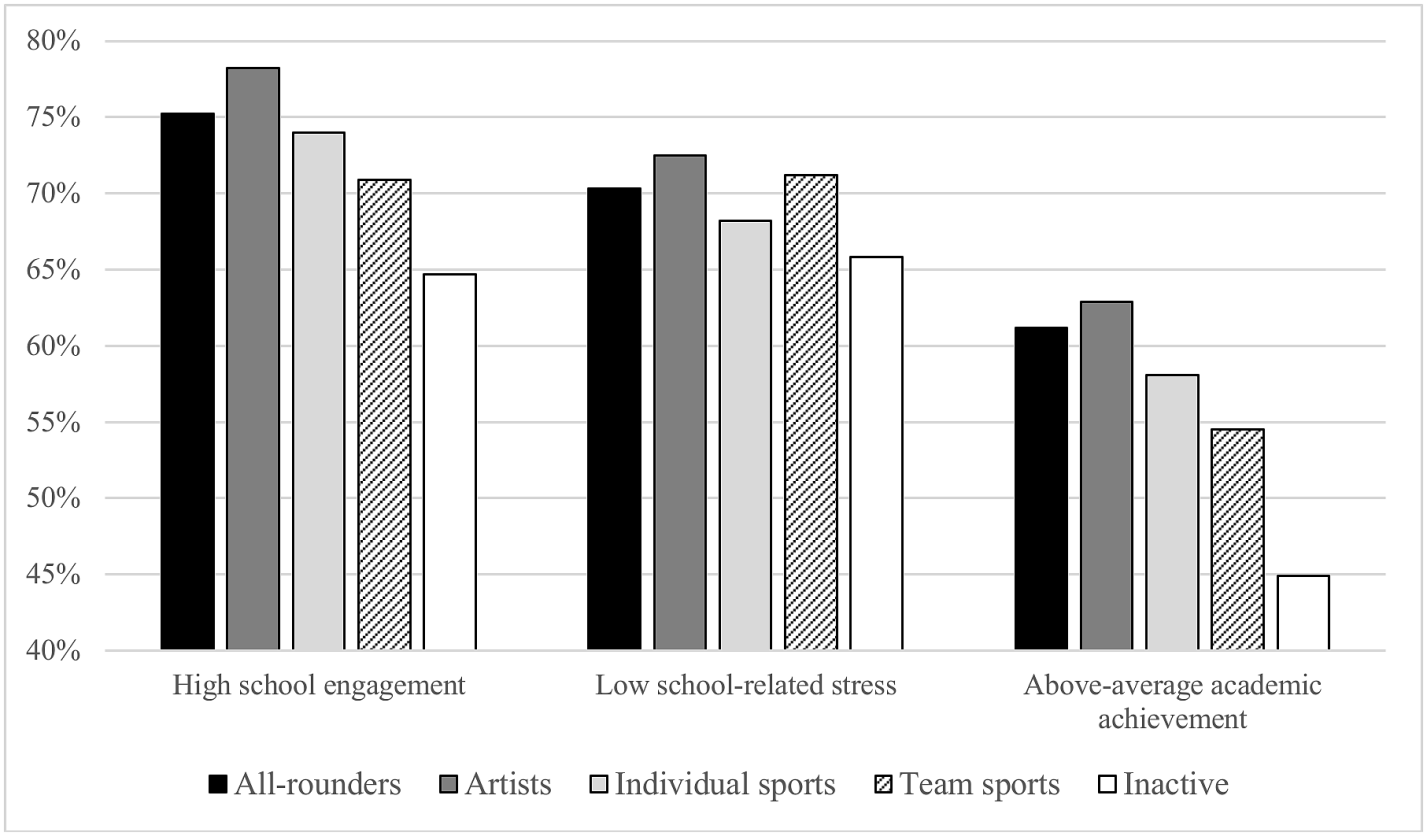
Percentages of adolescents, per OLTA cluster, who liked school, felt no or only a little pressure from schoolwork and rated their achievement as above-average (total sample, n = 10,483).

Independently of the affiliation to an OLTA cluster, the youngest age category and girls reported higher school engagement (*p*<0.001), lower levels of school-related stress (*p* = 0.018 for gender and *p*<0.001 for age difference) and better academic achievement (*p*<0.001) than the two older categories and boys, respectively. Nearly every other girl also had a non-familial person to help her with schoolwork, while in boys this was only one of three. The prevalence rates of school engagement, school-related stress and academic achievement—the three HBSC mandatory education-related items—in separate OLTA clusters are shown in Fig 1.

Table 2 presents odds ratios (OR) and 95% confidence intervals (CI) for the associations of the dichotomized participation variables with the school engagement, school-related stress, academic achievement and school support outside the family. Involvement in any OLTA was found to be significantly associated with all four explored education-related outcomes, even after adjustment for gender and age (Model 2). The strongest association was observed for academic achievement. Active adolescents had 1.81-times higher odds to self-report their achievement as good or very good. Last, we tested the interaction effects of gender and age. No interaction by gender was found to be statistically significant (S2 Table). We observed a statistically significant interaction of participating in OLTA with age for getting support outside the family, with 13-year-olds being more likely to get such support when involved in at least one OLTA than 15-year-olds (OR = 1.67, CI = 1.16–2.41). This question was not asked to 11-years-olds.

**Table 2:** Association of dichotomized participation variables with education-related outcomes: odds ratios and 95% confidence intervals for active vs. inactive adolescents (reference category).

	High school engagement	Low school-related stress	Above-average academic achievement	School support outside family
	(a lot/a bit)	(not at all/little)	(good/very good)	(peer and/or adult)
*Model 1 (univariable)*				
≥1 activity vs. inactive	1.61 (1.45–1.79)**	1.25 (1.13–1.39)**	1.81 (1.64–2.00)**	1.29 (1.09–1.53)*
*Model 2 (adjusted for age and gender)*				
≥1 activity vs. inactive	1.55 (1.39–1.72)**	1.20 (1.08–1.36)*	1.81 (1.64–2.00)**	1.38 (1.16–1.65)**

* p < 0.01,

** p < 0.001;

the item on school support outside family was present only in one questionnaire version for 13-year-olds and one version for 15-year-olds (n = 3,374).

Table 3 shows the results of the logistic regressions using clusters of OLTA as independent variables, with the inactive cluster as the reference category. Members of all active clusters were more likely to like school and rate their academic achievement as good or very good. The strongest associations with both outcomes were observed for ‘artists’, and the weakest for those participating only in team sports. An association with lower levels of school-related stress was observed in all active clusters of OLTA, except for the ‘individual sports’ cluster. Likewise, apart from the ‘team sports’ cluster the three remaining active clusters were more likely to acquire someone outside family to help them with schoolwork than inactive adolescents.

**Table 3:** Association of participation in organized leisure-time activities (clusters of activity pattern) with education-related outcomes: odds ratios and 95% confidence intervals for active vs. inactive adolescents (inactive cluster is the reference category).

	High school engagement	Low school-related stress	Above-average academic achievement	School support outside family
	(a lot/a bit)	(not at all/little)	(good/very good)	(peer and/or adult)
*Model 1 (univariable)*				
All-rounders	1.65 (1.46–1.87)***	1.23 (1.09–1.39)**	1.94 (1.73–2.17)***	1.55 (1.27–1.90)***
Artists	1.96 (1.70–2.26)***	1.37 (1.20–1.57)***	2.08 (1.83–2.37)***	1.62 (1.30–2.02)***
Individual sports	1.56 (1.34–1.81)***	1.12 (0.96–1.29)	1.70 (1.48–1.95)***	1.21 (0.96–1.54)
Team sports	1.33 (1.16–1.52)***	1.29 (1.13–1.78)***	1.47 (1.29–1.66)***	0.82 (0.66–1.03)
*Model 2 (adjusted for gender and age)*				
All-rounders	1.53 (1.35–1.73)***	1.15 (1.02–1.30)*	1.93 (1.72–2.17)***	1.66 (1.35–2.04)***
Artists	1.71 (1.48–1.98)***	1.34 (1.17–1.54)***	1.97 (1.73–2.25)***	1.50 (1.20–1.88)***
Individual sports	1.57 (1.34–1.82)***	1.09 (0.94–1.27)	1.73 (1.50–1.99)***	1.31 (1.03–1.67)*
Team sports	1.42 (1.24–1.63)***	1.23 (1.07–1.42)**	1.56 (1.37–1.78)***	0.99 (0.79–1.25)

* p < 0.05,

** p < 0.01,

*** p < 0.001;

the item on school support outside family was present only in one questionnaire version for 13-year-olds and one version for 15-year-olds (n = 3,374).

**Fig 2:**
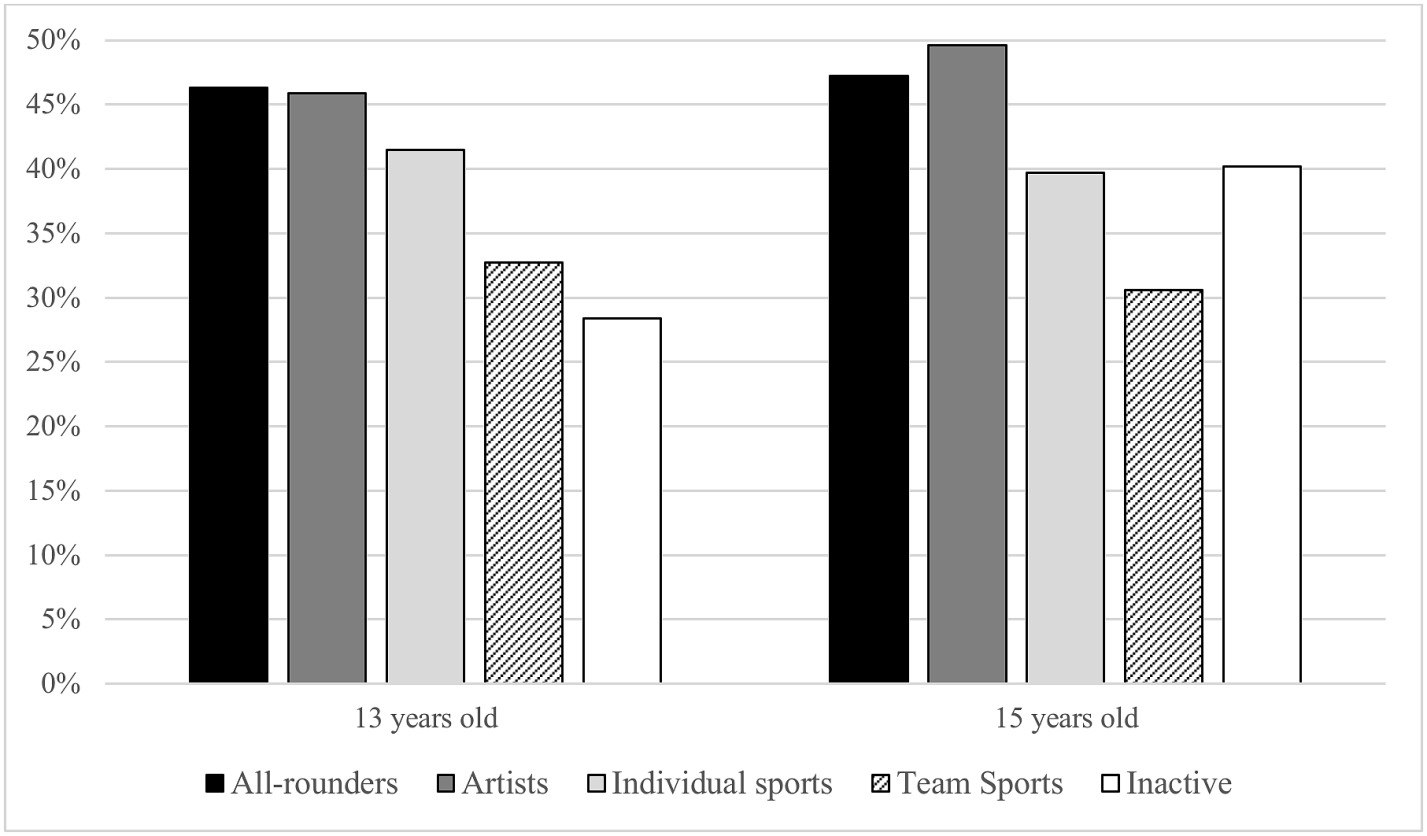
Percentages of 13- and 15-year old adolescents, per OLTA cluster, having someone outside their family to support them with schoolwork (n = 3,374); the question was not asked to 11-year-olds.

The interactions of OLTA clusters with gender were not significant (S3 Table). The interaction effects of the OLTA clusters with age were not statistically significant in terms of the overall variable p-value. However, after splitting the active participants into separate clusters, we found an interaction of age with ‘all-rounders’ (OR = 1.58, CI = 1.03–2.41), ‘individual sports’ (OR = 1.78, CI = 1.08–2.93) and ‘team sports’ (OR = 1.83, CI = 1.15–2.93) regarding support with schoolwork outside the family (S3 Table). Compared with their inactive counterparts, in all three mentioned clusters the respondents aged 13 were more likely to be supported with schoolwork by someone outside the family than those aged 15 (Fig 2).

## Discussion

We found that participation in OLTA was associated with higher school engagement, lower levels of school-related stress and better academic achievement. Nevertheless, the associations partly differed by specific OLTA patterns. Adolescents involved in more activities at the same time showed, in general, more positive school-related outcomes than non-involved adolescents and also than those involved only in sports, though this difference was not statistically significant. Moreover, OLTA participants were more likely to have someone outside the family to help them with schoolwork, with this association being weaker in 15-year-olds.

Adolescents involved in any type of OLTA reported better than their inactive peers on all four education-related outcomes included in this study. This finding is in line with previous research, predominantly from the USA, linking OLTA to students’ improved school performance and higher bonding to school [10, 14]. It might thus be inferred that the mechanism underlying participation in OLTA, as suggested for the US setting, i.e. utilizing contextual developmental assets to nurture individual strengths [37, 38], might also be applicable to the European school and leisure environment.

We also found that different patterns of OLTA participation (clusters of OLTA) had varying associations with school performance and school engagement. Adolescents participating only in sports, either team and/or individual ones, reported performing better on all the analysed education-related outcomes than the inactive adolescents. This is in agreement with the recent findings linking moderate physical activity throughout adolescence to better cognitive performance at young adulthood [39] and, more specifically, sports participation to better mathematic performance [40]. Moreover, the adolescents participating in more than just in sports, i.e. members of ‘artists’ or ‘all-rounders’ clusters reported even better outcomes than those participating only in sports. This accords with the results of one of the few European studies on this topic, by Metsäpelto & Pulkkinen [41], who observed stronger association between performing arts and higher academic working skills and academic attainments compared with sportsmen.

Considering that over a half of artists and a vast majority of all-rounders participated in two or more activities, our findings support the assumption that the more activities adolescents are involved in, the better it is for them [9, 22, 23]. Involvement in a variety of distinct out-of-school contexts exposes adolescents to a range of different situations, challenges and diverse persons, which both enriches their social networks and lets them adopt skills helpful for coping with their school life [42]. The finding is also remarkable in light of previous research on OLTA and health [31], which contrarily showed the strongest association with physical and mental health in youth participation only in sports. This clearly underlines the hypothesised uniqueness of the link between different activities (pattern of activities in our case) and various youth developmental indicators [30, 43, 44].

All-rounders, artists and individual sports participants were also more likely to acquire non-familial support for schoolwork. However, we observed an interaction effect of age on this outcome, with 13-year-olds being more likely to get such support than 15-year-olds when affiliated to one of the OLTA clusters (except for the artists, in which the association did not differ by age). This was probably due to the noticeable increase in the number of inactive adolescents with such support between 13 and 15 years of age. In the Czech Republic, the 9^th^ grade (15-year-olds) is the last one before transition to the secondary school. It appears that inactive youth, who generally do worse in school, try to compensate for their poor performance through receiving extracurricular tutoring in order to increase their chance to get accepted to the desired school. This would be in line with Himelfarb et al. [12], who found that the frequency of academic tutoring was associated with worse school grades. Moreover, it could be a cause of the weaker associations between OLTA participation and school support outside the family in older adolescents.

### Strengths and limitations

The most important strength of this study is its large and representative sample. Furthermore, this study was based on the well-established and recognized HBSC study, with a strong methodological background regarding data collection procedures and construction of the questionnaire, which is subject to regular revisions by an international expert team.

Our findings need to be interpreted in light of some limitations, however. First, the cross-sectional design hinders the potential for conclusions on causality between participation in OLTA and enhanced education-related outcomes. Second, we used self-reported data, which might be more prone to be biased. However, the mandatory school-related items have been included in the HBSC study since 1985/1986 (school-related stress since 1993/1994) and widely used [32, 45–47]. The academic achievement item has been shown to be a valid and reliable question [48, 49] and the validation works on the two remaining items were ongoing at the time of the last 2013/2014 HBSC survey [32]. Last, we did not collect information on other dimensions of OLTA, including intensity, duration and engagement, which are known to have unique links to developmental outcomes [50] and might have had affected the associations found.

### Implications

The findings of this study demonstrate the associations between involvement in OLTA and improved education-related outcomes in adolescents in a European context and confirm findings from the USA. The present results highlight the previously proposed benefits of participation in various activities concurrently, which leads to more positive outcomes. The study also shows that OLTA participation provides youth with opportunities to meet new people possibly influential for their school performance. It can serve as a form of ‘resource compensation’ and reduce the achievement gap between less and more academically successful youth [51], in younger age categories in particular. As a result, participation in OLTA might contribute to a prospective academic career [52] and subsequently also to the work-career development of current adolescents. Future research should focus on analysing the causal relationship between OLTA and education-related outcomes in Europe, because, to the best of our knowledge, such work has not yet been conducted.

### Conclusion

The associations between participation in OLTA and better education-related outcomes observed in this study indicate that previously suggested benefits of OLTA for adolescents’ education might also apply to the European context. Our findings may serve as a cue that OLTA improve the relationship to school and the school performance in youth.

## Supporting Information

S1 Table Tables A & B. Associations of OLTA participation with education-related outcomes adjusted for SES.
(DOCX)

S2 Table Associations of dichotomized OLTA variable with education-related outcomes including interactions with gender and age. (DOCX)

S3 Table Associations of OLTA clusters with education-related outcomes including interactions with gender and age. (DOCX)
