# Supplementary material for: Is Participation in Organized Leisure-Time Activities Associated with School Performance in Adolescence?
Source: PLoS One. 2016 Apr 13;11(4):e0153276. doi: 10.1371/journal.pone.0153276 (PMC4830594; doi:10.1371/journal.pone.0153276)
Supplement: S1 Table — Associations of OLTA participation with education-related outcomes adjusted for SES. (DOCX) [file pone.0153276.s001.docx]

Table A. Association of dichotomized participation variables with education-related outcomes: odds ratios and 95% confidence intervals for active vs. inactive adolescents (reference category)

|  | High school engagement  (a lot/a bit) | Low school-related stress  (not at all/little) | | Above-average academic achievement  (good/very good) | School support outside family  (peer and/or adult) |
| --- | --- | --- | --- | --- | --- |
| Model 1 (univariable) | | |  | | |
| ≥1 activity vs. inactive | **1.61 (1.45-1.79)***** | **1.25 (1.13-1.39)***** | | **1.81 (1.64-2.00)***** | **1.29 (1.09-1.53)**** |
| Model 2 (adjusted for age and gender) | | |  | | |
| ≥1 activity vs. inactive | **1.55 (1.39-1.72)***** | **1.20 (1.08-1.36)**** | | **1.81 (1.64-2.00)***** | **1.38 (1.16-1.65)***** |
| Model 3 (adjusted for age, gender and socioeconomic status) | | | | | |
| ≥1 activity vs. inactive | **1.52 (1.37-1.70)***** | **1.21 (1.08-1.34)***** | | **1.76 (1.59-1.95)***** | **1.36 (1.14-1.62)***** |

* *p* < 0.05, ** *p* < 0.01, *** *p* < 0.001; *the* *item on school support outside family was present only in one questionnaire version for 13-year-olds and one version for 15-year-olds (n = 3,374).*

Table B. Association of participation in organized leisure-time activities (clusters of activity pattern) with education-related outcomes: odds ratios and 95% confidence intervals for active vs. inactive adolescents (inactive cluster is the reference category)

|  | High school engagement  (a lot/a bit) | Low school-related stress  (not at all/little) | Above-average academic achievement  (good/very good) | School support outside family  (peer and/or adult) |
| --- | --- | --- | --- | --- |
| Model 1 (univariable) | | | |  |
| All-rounders | **1.65 (1.46-1.87)***** | **1.23 (1.09-1.39)**** | **1.94 (1.73-2.17)***** | **1.55 (1.27-1.90)***** |
| Artists | **1.96 (1.70-2.26)***** | **1.37 (1.20-1.57)***** | **2.08 (1.83-2.37)***** | **1.62 (1.30-2.02)***** |
| Individual sports | **1.56 (1.34-1.81)***** | 1.12 (0.96-1.29) | **1.70 (1.48-1.95)***** | 1.21 (0.96-1.54) |
| Team sports | **1.33 (1.16-1.52)***** | **1.29 (1.13-1.78)***** | **1.47 (1.29-1.66)***** | 0.82 (0.66-1.03) |
| Model 2 (adjusted for gender and age) | | | |  |
| All-rounders | **1.53 (1.35-1.73)***** | **1.15 (1.02-1.30)*** | **1.93 (1.72-2.17)***** | **1.66 (1.35-2.04)***** |
| Artists | **1.71 (1.48-1.98)***** | **1.34 (1.17-1.54)***** | **1.97 (1.73-2.25)***** | **1.50 (1.20-1.88)***** |
| Individual sports | **1.57 (1.34-1.82)***** | 1.09 (0.94-1.27) | **1.73 (1.50-1.99)***** | **1.31 (1.03-1.67)*** |
| Team sports | **1.42 (1.24-1.63)***** | **1.23 (1.07-1.42)**** | **1.56 (1.37-1.78)***** | 0.99 (0.79-1.25) |
| Model 3 (adjusted for age, gender and socioeconomic status) | | | | |
| All-rounders | **1.52 (1.34-1.72)***** | **1.16 (1.03-1.32)*** | **1.87 (1.67-2.11)***** | **1.63 (1.33-2.00)***** |
| Artists | **1.68 (1.45-1.95)***** | **1.35 (1.17-1.55)***** | **1.91 (1.67-2.18)***** | **1.47 (1.17-1.85)***** |
| Individual sports | **1.53 (1.31-1.79)***** | 1.10 (0.95-1.28) | **1.70 (1.47-1.96)***** | **1.28 (1.00-1.63)*** |
| Team sports | **1.40 (1.22-1.61)***** | **1.23 (1.07-1.42)**** | **1.54 (1.35-1.75)***** | 0.99 (0.78-1.24) |

* *p* < 0.05, ** *p* < 0.01, *** *p* < 0.001; *the* *item on school support outside family was present only in one questionnaire version for 13-year-olds and one version for 15-year-olds (n = 3,374).*
