# Supplementary material for: Is Participation in Organized Leisure-Time Activities Associated with School Performance in Adolescence?
Source: PLoS One. 2016 Apr 13;11(4):e0153276. doi: 10.1371/journal.pone.0153276 (PMC4830594; doi:10.1371/journal.pone.0153276)
Supplement: S2 Table — (DOCX) [file pone.0153276.s002.docx]

Table. Association of dichotomized participation variables with education-related outcomes: odds ratios and 95% confidence intervals for active vs. inactive adolescents (reference category)

|  | High school engagement  (a lot/a bit) | Low school-related stress  (not at all/little) | | Above-average academic achievement  (good/very good) | School support outside family  (peer and/or adult) |
| --- | --- | --- | --- | --- | --- |
| Model 1 (univariable) | | |  | | |
| ≥1 activity vs. inactive | **1.61 (1.45-1.79)***** | **1.25 (1.13-1.39)***** | | **1.81 (1.64-2.00)***** | **1.29 (1.09-1.53)**** |
| Model 2 (adjusted for age and gender) | | |  | | |
| ≥1 activity vs. inactive | **1.55 (1.39-1.72)***** | **1.20 (1.08-1.36)**** | | **1.81 (1.64-2.00)***** | **1.38 (1.16-1.65)***** |
| Model 3 (including interaction with gender, adjusted for age) | | | | | |
| ≥1 activity vs. inactive | **1.65 (1.42-1.91)***** | 1.16 (1.00-1.34) | | **1.84 (1.60-2.11)***** | **1.37 (1.09-1.72)**** |
| Gender M vs. F | **0.74 (0.62-0.90)**** | 1.04 (0.86-1.25) | | **0.78 (0.65-0.93)**** | **0.49 (0.36-0.67)***** |
| ≥1 activity M vs. F | 0.88 (0.72-1.09) | 1.09 (0.88-1.34) | | 0.97 (0.79-1.18) | 1.02 (0.72-1.45) |
| Model 4 (including interaction with age, adjusted for gender) | | | | | |
| ≥1 activity vs. inactive | **1.55 (1.33-1.81)***** | **1.29 (1.10-1.50)**** | | **1.98 (1.71-2.31)***** | 1.14 (0.92-1.42) |
| Age 11- vs 15-yrs | **1.54 (1.20-1.97)***** | **1.49 (1.16-1.91)**** | | 1.21 (0.96-1.52) | N/A |
| Age 13- vs 15-yrs | 1.18 (0.95-1.46) | 1.10 (0.89-1.36) | | 1.10 (0.89-1.36) | **0.60 (0.43-0.83)**** |
| ≥1 activity 11 vs 15 | 1.01 (0.76-1.33) | 0.98 (0.74-1.29) | | 0.89 (0.67-1.14) | N/A |
| ≥1 activity 13 vs 15 | 0.91 (0.77-1.26) | 0.82 (0.64-1.05) | | 0.82 (0.65-1.04) | **1.67 (1.16-2.41)**** |

* *p* < 0.05, ** *p* < 0.01, *** *p* < 0.001; *M - males; F - females; yrs – years old* *the* *item on school support outside family was present only in one questionnaire version for 13-year-olds and one version for 15-year-olds (n = 3,374).*
