# Supplementary material for: Is Participation in Organized Leisure-Time Activities Associated with School Performance in Adolescence?
Source: PLoS One. 2016 Apr 13;11(4):e0153276. doi: 10.1371/journal.pone.0153276 (PMC4830594; doi:10.1371/journal.pone.0153276)
Supplement: S3 Table — (DOCX) [file pone.0153276.s003.docx]

Table A. Association of participation in organized leisure-time activities (clusters of activity pattern) with education-related outcomes: odds ratios and 95% confidence intervals for active vs. inactive adolescents (inactive cluster is the reference category)

|  | High school engagement  (a lot/a bit) | Low school-related stress  (not at all/little) | Above-average academic achievement  (good/very good) | School support outside family  (peer and/or adult) |
| --- | --- | --- | --- | --- |
| Model 1 (univariable) | | | |  |
| All-rounders | **1.65 (1.46-1.87)***** | **1.23 (1.09-1.39)**** | **1.94 (1.73-2.17)***** | **1.55 (1.27-1.90)***** |
| Artists | **1.96 (1.70-2.26)***** | **1.37 (1.20-1.57)***** | **2.08 (1.83-2.37)***** | **1.62 (1.30-2.02)***** |
| Individual sports | **1.56 (1.34-1.81)***** | 1.12 (0.96-1.29) | **1.70 (1.48-1.95)***** | 1.21 (0.96-1.54) |
| Team sports | **1.33 (1.16-1.52)***** | **1.29 (1.13-1.78)***** | **1.47 (1.29-1.66)***** | 0.82 (0.66-1.03) |
| Model 2 (adjusted for gender and age) | | | |  |
| All-rounders | **1.53 (1.35-1.73)***** | **1.15 (1.02-1.30)*** | **1.93 (1.72-2.17)***** | **1.66 (1.35-2.04)***** |
| Artists | **1.71 (1.48-1.98)***** | **1.34 (1.17-1.54)***** | **1.97 (1.73-2.25)***** | **1.50 (1.20-1.88)***** |
| Individual sports | **1.57 (1.34-1.82)***** | 1.09 (0.94-1.27) | **1.73 (1.50-1.99)***** | **1.31 (1.03-1.67)*** |
| Team sports | **1.42 (1.24-1.63)***** | **1.23 (1.07-1.42)**** | **1.56 (1.37-1.78)***** | 0.99 (0.79-1.25) |
| Model 3 (including interaction with gender, adjusted for age) | | | | |
| All-rounders | **1.68 (1.41-2.01)***** | 1.15 (0.97-1.35) | **2.00 (1.71-2.35)***** | **1.49 (1.13-1.94)**** |
| Artists | **1.72 (1.43-2.07)***** | **1.29 (1.08-1.53)**** | **1.97 (1.67-2.32)***** | **1.53 (1.16-2.04)**** |
| Individual sports | **1.63 (1.30-2.05)***** | 0.99 (0.80-1.22) | **1.63 (1.33-1.90)***** | 1.30 (0.94-1.82) |
| Team sports | **1.40 (1.11-1.78)**** | 1.09 (0.87-1.36) | **1.41 (1.14-1.75)**** | 0.95 (0.67-1.36) |
| Gender M vs. F | **0.74 (0.62-0.90)**** | 1.04 (0.86-1.25) | **0.78 (0.65-0.93)**** | **0.49 (0.36-0.67)***** |
| All-rounders M vs. F | 0.84 (0.65-1.07) | 1.02 (0.80-1.30) | 0.93 (0.74-1.16) | 1.28 (0.85-1.93) |
| Artists M vs. F | 1.03 (0.76-1.39) | 1.10 (0.81-1.48) | 1.01 (0.76-1.32) | 0.89 (0.54-1.46) |
| Ind. sports M vs. F | 0.92 (0.68-1.26) | 1.23 (0.91-1.65) | 1.12 (0.85-1.48) | 1.03 (0.63-1.68) |
| Team sports M vs. F | 0.99 (0.74-1.33) | 1.23 (0.92-1.64) | 1.16 (0.88-1.52) | 1.10 (0.68-1.77) |
| Model 4 (including interaction with age, adjusted for gender) | | | | |
| All-rounders | **1.63 (1.33-1.99)***** | 1.17 (0.96-1.42) | **2.17 (1.79-2.63)***** | **1.41 (1.07-1.85)*** |
| Artists | **1.50 (1.20-1.87)***** | **1.35 (1.08-1.68)**** | **2.06 (1.66-2.54)***** | 1.35 (1.00-1.83) |
| Individual sports | **1.75 (1.39-2.21)***** | **1.29 (1.03-1.62)*** | **2.02 (1.63-2.51)***** | 1.05 (0.76-1.45) |
| Team sports | **1.37 (1.12-1.68)**** | **1.37 (1.11-1.69)**** | **1.71 (1.40-2.07)***** | 0.79 (0.58-1.06) |
| Age 11- vs 15-yrs | **1.53 (1.20-1.96)***** | **1.49 (1.16-1.91)**** | 1.20 (0.96-1.51) | N/A |
| Age 13- vs 15-yrs | 1.18 (0.95-1.46) | 1.10 (0.89-1.36) | 1.10 (0.89-1.35) | **0.60 (0.43-0.83)**** |
| All-rounders 11- vs 15-yrs | 0.93 (0.67-1.27) | 1.01 (0.73-1.38) | 0.83 (0.62-1.12) | N/A |
| Artists 11- vs 15-yrs | 1.39 (0.96-2.01) | 1.20 (0.84-1.71) | 0.96 (0.69-1.33) | N/A |
| Ind. sports 11- vs 15-yrs | 0.87 (0.58-1.30) | 0.88 (0.59-1.29) | 0.81 (0.57-1.16) | N/A |
| Team sports 11- vs 15-yrs | 0.92 (0.65-1.31) | 0.85 (0.59-1.21) | 0.87 (0.63-1.20) | N/A |
| All-rounders 13- vs 15-yrs | 0.91 (0.68-1.23) | 0.93 (0.70-1.24) | 0.81 (0.61-1.07) | **1.58 (1.03-2.41)*** |
| Artists 13- vs 15-yrs | 1.10 (0.79-1.54) | 0.82 (0.60-1.34) | 0.87 (0.63-1.18) | 1.42 (0.89-2.26) |
| Ind. sports 13- vs 15-yrs | 0.79 (0.55-1.12) | **0.67 (0.48-0.94)*** | **0.72 (0.52-1.00)*** | **1.78 (1.08-2.93)*** |
| Team sports 13- vs 15-yrs | 1.16 (0.85-1.59) | 0.81 (0.60-1.11) | 0.83 (0.62-1.12) | **1.83 (1.15-2.93)*** |

* *p* < 0.05, ** *p* < 0.01, *** *p* < 0.001; *M - males; F - females; yrs – years old,* *the* *item on school support outside family was present only in one questionnaire version for 13-year-olds and one version for 15-year-olds (n = 3,374).*
